# The Envelope (E) Protein of SARS-CoV-2 as a Pharmacological Target

**DOI:** 10.3390/v15041000

**Published:** 2023-04-19

**Authors:** Teresa Santos-Mendoza

**Affiliations:** Laboratory of Transcriptomics and Molecular Immunology, Instituto Nacional de Enfermedades Respiratorias Ismael Cosío Villegas, Mexico City 14080, Mexico; tsantos@iner.gob.mx

**Keywords:** coronavirus E protein, PDZ-dependent interactions, antivirals

## Abstract

The COVID-19 pandemic caused by the SARS-CoV-2 virus is still a global health concern. Several spike (S) protein-based vaccines have been developed that efficiently protect the human population against severe forms of COVID-19. However, some SARS-CoV-2 variants of concern (VOCs) have emerged that evade the protective effect of vaccine-induced antibodies. Therefore, efficient and specific antiviral treatments to control COVID-19 are indispensable. To date, two drugs have been approved for mild COVID-19 treatment; nevertheless, more drugs, preferably broad-spectrum and ready-to-use therapeutic agents for new pandemics, are needed. Here, I discuss the PDZ-dependent protein-protein interactions of the viral E protein with host proteins as attractive alternatives for the development of antivirals against coronavirus.

## 1. Introduction

In the past 20 years, three highly pathogenic coronaviruses (CoVs) have emerged as threats to humans: severe acute respiratory syndrome coronavirus (SARS-CoV-1) in 2002; Middle East respiratory syndrome coronavirus (MERS) in 2012; and the currently circulating SARS-CoV-2, the causative agent of the recent COVID-19 pandemic [1]. These three highly pathogenic CoVs belong to the β-coronavirus genera and can cause severe airway infection and acute respiratory distress syndrome (ARDS). CoVs are enveloped viruses with a single-stranded positive RNA genome that encodes four structural proteins: nucleocapsid (N), membrane (M), S, and E [2]. The S protein of SARS-CoVs recognizes cell receptors to initiate infection; thus, many efforts toward vaccine development, as well as therapeutic and diagnostic tools, have focused on this protein. Several S protein-based vaccines have been developed, including those based on mRNA or adenoviral vectors, which efficiently protect the human population against severe forms of COVID-19 [3]. However, some SARS-CoV-2 variants of concern (VOCs) evade the protective effects of vaccine-induced antibodies. In this scenario, efficient antiviral treatments, and the two currently used to control COVID-19, are indispensable. Many experimental efforts have resulted in a variety of compounds, including small molecule inhibitors [4,5], bioactive natural products, and/or traditional medicine products that have shown effectiveness against SARS-CoV-2 [6,7,8]. It is necessary to deepen the study of these compounds until an adequate drug design is obtained. This study focused on the E protein of highly pathogenic CoVs, with an emphasis on SARS-CoVs as a potential target for designing novel therapeutic molecules against COVID-19 and their possible use as broad-spectrum antivirals.

## 2. Highly Pathogenic Coronaviruses

SARS-CoV-1 was first detected in China in late 2002 and spread to 29 other countries, killing more than 800 individuals with almost 9000 confirmed cases and an average mortality of 10%. This public health emergency prompted research on CoVs. Among the distinct lines of research, scientists noticed the existence of a large reservoir of SARS-CoV-1-like viruses in diverse animals, with bats being the most important, and warned about the possibility of SARS-CoV-1 re-emergence or the emergence of other novel CoVs, suggesting the need for preparedness for other outbreaks caused by CoVs [1,9,10]. As predicted, another highly pathogenic CoV, MERS, emerged by the end of 2012. A close relative of SARS-CoV-1, MERS has a threefold mortality rate which fortunately was less efficient in human-to-human transmission. MERS remained limited to the Middle East, causing 858 deaths out of 2494 confirmed infections [2,9]. Now, SARS-CoV-2 has been circulating worldwide for three years, representing the deadliest respiratory disease pandemic since the Spanish Flu of 1918, with almost seven million deaths worldwide (https://covid19.who.int/ accessed on 14 April 2023) [9,11]. Among the diverse recommendations of public health organizations since the emergence of SARS-CoV-1, the development and stockpiling of drugs that target a wide range of viral pathogens (broad-spectrum antiviral drugs) have been a constant recommendation that, unfortunately, has not been adequately addressed [12]. The current pandemic is a reminder that antivirals against SARS-CoVs should be developed and that we are still unprepared for the next outbreaks that will undoubtedly occur.

## 3. Antivirals against Coronaviruses

For years, therapeutic approaches to treat viral infections have been focused on targeting viral components; for example, the proteins that recognize cell receptors to initiate infection, the RNA-dependent RNA polymerases, or other viral enzymes [13]. Antivirals targeting viral components are highly susceptible to drug resistance owing to the rapidly evolving viral genome. This is exemplified by the emergence of influenza strains that are resistant to adamantanes, inhibitors of the M2 ionic channel, and oseltamivir, an inhibitor of neuraminidase [14]. Moreover, influenza strains with dual adamantane-oseltamivir resistance have been reported [15]. The risk of developing resistance is particularly important when using drugs targeting only one viral component; therefore, additional approaches for antiviral development are necessary.

Another strategy for developing antivirals has also been proposed. Viruses take over the host cellular machinery for replication and dissemination. Therefore, targeting the host elements essential for the viral life cycle is an attractive alternative for the development of antivirals [13,16]. Distinct experimental approaches, including computational, proteomic, and genetic analyses, have been used to identify these essential virus–host interactions. Based on this strategy, for example, silencing or inhibition of the host C-terminal Src kinase (Csk) was found to reduce hepatitis C virus (HCV) replication [17,18].

Since the emergence of SARS-CoV-1, efforts to identify antiviral molecules have shed light on the strategies for developing specific or pan-coronavirus inhibitors. Papain-like (PL) or main (3CL) CoVs proteases have emerged as efficient antiviral targets [10,19,20]. In contrast, as an example of host targeting, the inhibition of p38 mitogen-activated protein kinase (p38 MAPK) has shown an effective antiviral function [21,22].

The two drugs currently approved for mild COVID-19 treatment, Molnupiravir and Nirmatrelvir, belong to the first type of antivirals that target viral components. Molnupiravir is a prodrug that is metabolized to the cytidine nucleoside analog beta-D-N4-hydroxycytidine (NHC), which, upon phosphorylation, generates the nucleotide analog NHC-TP. During replication, NHC-TP is incorporated into the SARS-CoV-2 RNA by viral RNA polymerase instead of cytidine or uridine nucleotides, causing permanent mutations in the viral genome. Hence, molnupiravir inhibits replication by interfering with the viral RNA polymerase [19,23].

In contrast, nirmatrelvir inhibits the SARS-CoV-2 main protease Mpro. It was obtained by improving a previous inhibitor of SARS-CoV-1. Mpro is required for post-translational proteolytic processing of two polyproteins translated from the open reading frame (ORF) 1a and ORF1b to generate nonstructural viral proteins (NSPs). Therefore, nirmatrelvir may play a key role in blocking viral transcription and replication [19,22,23]. Nirmatrelvir is administered with ritonavir, which increases its bioavailability (Table 1).

Remdesivir, first designed for the prevention of Ebola infection, is another nucleotide analog that targets viral RNA polymerase and was approved for COVID-19 treatment in May 2020 [24]. However, the World Health Organization removed it from the list of effective drugs against COVID-19 in November 2020 and reported that, based on a large number of clinical trials, there was no evidence that remdesivir improved survival and other outcomes in patients (https://www.who.int/news-room/feature-stories/detail/who-recommends-against-the-use-of-remdesivir-in-covid-19-patients, accessed on 6 March 2023).

**Table 1:** Description of FDA-approved antiviral drugs. GFR: glomerular filtration rate.

Antiviral	Indications and Dosage	Interactions with Other Drugs	Effectiveness	References
Molnupiravir (Lagevrio)	Outpatient treatment of mild to moderate COVID-19 in adults (≥18 years). Initiate as soon as possible and within 5 days of symptom onset.	No drug interactions with molnupiravir have yet been identified.	Antiviral activity against SARS-CoV-2 in cell culture assays with a 50% effective concentration (EC50) ranging between 0.67 to 2.66 µM. NHC had similar activity against the variants including Alpha, Beta, Gamma, and Delta.	[23,25,26]
	Oral use: 200 mg capsules. The authorized dose is 800 mg every 12 h for 5 days.			
	Not recommended in severe renal impairment (eGFR < 30 mL/min).			
Nirmatrelvir-Ritonavir (Paxlovid)	Outpatient treatment of mild to moderate COVID-19 in adults and in children down to age 12 who weigh at least 40 kg. Initiate as soon as possible and within 5 days of symptom onset.	Of major concern, altered drug concentrations are contraindicated due to possible serious or life-threatening reactions.	Antiviral activity against SARS-CoV-2 Alpha, Beta, Gamma, Delta, and Lambda variants. Activity against SARS-CoV2 in A549-ACE2 cells is of 77.9 and a 90% effective concentration (EC90) of 215 nM.	[23,27]
	Oral use: 150 mg nirmatrelvir and 100 mg ritonavir.			
	The authorized dose is 300–100 mg every 12 h for 5 days			

Understanding the molecular interactions between SARS-CoV-2 and human cells, specifically protein–protein interactions (PPIs), will allow the identification of key host proteins hijacked by the virus for their essential role in its life cycle. This knowledge is the basis for developing strategies to target these essential host–virus interacting elements and, considering that the virus is less likely to mutate at such specific, essential interacting moieties, to reduce the emergence of drug resistance [13,22].

Since the emergence of SARS-CoV-2, efforts to identify virus–host PPIs have identified targets for drug design and repurposing. In a pioneer study, Gordon et al. (2020) cloned 26 out of 29 viral proteins and identified 332 host-interacting proteins, 66 of which were druggable targets of existing compounds, with 29 approved by the Federal Drug Administration (FDA) [28]. Among druggable host targets, proteins involved in processes such as trafficking, lipid remodeling and endoplasmic reticulum (ER) stress response, translation, and several innate immune signaling proteins related to the interferon pathway were found as targets of diverse viral proteins. For example, the translation regulatory protein eIF4H was identified as the target of the viral NSP9 protein. Zotatifin is an inhibitor of protein biogenesis by targeting eIF4A, a partner of eIF4H in a translational functional complex. This compound showed a strong anti-SARS-CoV-2 effect in vitro and is currently in clinical trials for cancer treatment and, more recently, also for COVID-19 treatment based on in vitro and in silico models [28,29,30].

Moreover, in another study, Gordon et al. (2020b) extended their analyses to compare the virus–host PPIs of three highly pathogenic CoVs and complemented them with functional assays. In this manner, the authors identified conserved interactions across the three highly pathogenic CoVs, as well as the cellular processes affected, pointing to the design of pan-coronavirus antivirals [31]. Such integrative studies represent new approaches in drug design from which pan-viral inhibitors can emerge, which may help prepare for future outbreaks and/or pandemics.

## 4. The Envelope (E) Protein of SARS-CoVs

**Figure 1:**
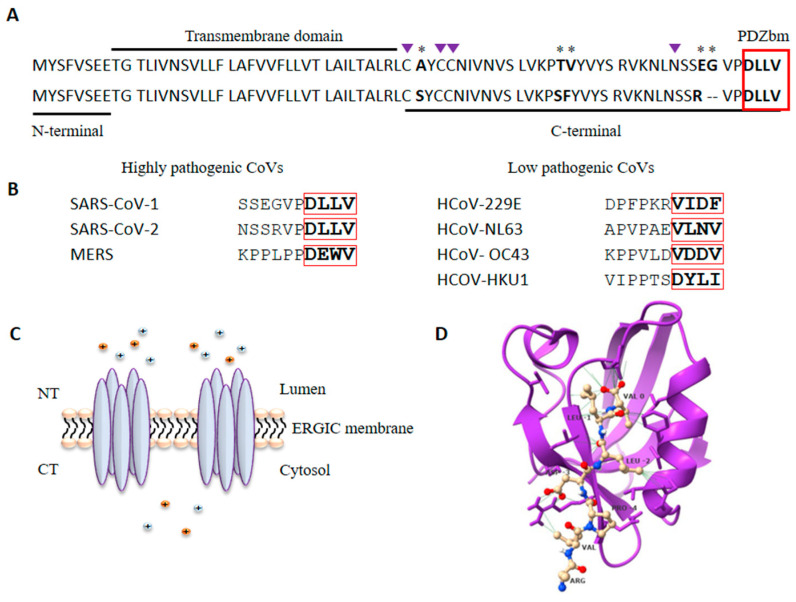
The E protein of SARS-CoV-1 and SARS-CoV-2 are 96% similar. (**A**) Alignment of the E protein sequence of SARS-CoV-1 (upper sequence) and SARS-CoV-2 (lower sequence); the three domains and the PDZbm (red box) are shown. * show amino acid differences and deletions between both proteins. Purple triangles indicate residues susceptible to posttranslational modification. (**B**) Alignment of the last ten amino acids of the highly pathogenic (left) and low pathogenic human CoVs showing the PDZbm. (**C**) Pentameric homo-oligomerization of the E protein forms an ion channel or viroporin. (**D**) Structure of the first PDZ domain of PALS1 in complex with the E protein of SARS-CoV-2 (PDB ID: 7NTK). Three-dimensional structure of PDZ1 domain of PALS1 (purple) in complex with the PDZbm of the E protein of SARS-CoV-2 (yellow); Figure is drawn with ChimeraX 1.2.5. (University of California at San Francisco, San Francisco, CA, USA).

These three highly pathogenic CoVs belong to the β-coronavirus genera. Phylogenetic analyses locate MERS more distant from SARS-CoV-1 and SARS-CoV-2, which share approximately 85% sequence similarity. The CoVs genome encodes four structural proteins: spike (S), membrane (M), nucleocapsid (N), and envelope (E) [9,20]. The CoVs E protein is a transmembrane protein of 75–109 amino acids (aa) and 8–12 KDa present in low quantities in viral particles but highly expressed in infected cells. It can be divided into three domains: a short N-terminal hydrophilic domain, a transmembrane domain, and a C-terminal domain containing diverse motifs for glycosylation, palmitoylation, and PPI (Figure 1A) [32,33]. SARS-CoV-1 E protein is susceptible to N-glycosylation at the N66 residue conserved in SARS-CoV-2, although the functional outcome of such modification is still unknown [33,34]. The conserved cysteine residues C40, C43 and C44 can be palmitoylated; this modification might regulate E trafficking and association with lipid rafts [33,35,36].

The E protein maintains the viral particle structure by interacting with the M protein. The E protein can homo-oligomerize to form a cation-selective ion channel (IC) or viroporin under physiological conditions and is mainly located in the Golgi, ER, and ER-Golgi intermedium compartment (ERGIC) of infected cells, with the N-terminus towards the lumen and the C-terminus towards the cytosol, where it is involved in morphogenesis and viral assembly [32,37]. Ion channel formation appears to be one of the first and most important events in direct viral pathophysiology [38]. Many viruses express viroporins; for example, the protein M2 of the influenza A virus, whose activity is indispensable for viral RNA release from the endosome to the cytoplasm, is an ion channel and was the first discovered viroporin. Other viruses, such as HIV-1, RSV, and Hepatitis C, have been shown to express the viroporins Vpu, SH, and p7, respectively, which are relevant elements in the viral life cycle [33,39]. In general, viroporins are proteins with IC activity that interfere with different cell membranes, affecting membrane permeability, membrane potential, ionic gradients, and pH, among other features, with consequences for diverse cellular processes, such as trafficking, signal transduction, and apoptosis [39].

The E viroporin acts as a major trigger for the host inflammatory response by activating the NLRP3 (NOD-, LRR- and pyrin domain-containing protein 3) inflammasome, thus inducing interleukin (IL)-1β secretion, as well as tumor necrosis factor-alpha (TNFα) and IL-6, and is a determinant of pathogenicity [32,40]. Recombinant viruses with an E protein lacking ion channel activity are attenuated in mice with a considerable reduction in lung epithelial damage and inflammation and reduced mortality, despite similar viral replication in vivo compared to the wild-type virus [41]. Since the SARS-CoV-1 emergence, blockers of ion channel activity of the E protein have been shown to counteract virus-induced diseases [37]. Amantadine is a broad-spectrum viroporin inhibitor that was previously used to treat influenza A. Broad-spectrum antivirals are molecules with proven efficacy against distinct viruses, even from different viral families; this feature of such antivirals is particularly relevant when there is an urgency to manage emerging virus outbreaks, as was the case with SARS-CoV-2 [42]. Amantadine was assayed for its potential use in SARS-CoV-induced diseases; however, it showed only moderate inhibition of the E protein IC activity of CoVs [33,43]. In silico docking analyses of large sets of approved drugs have revealed several molecules with the potential to block the IC activity of the SARS-CoV E protein for potential use in humans, which need to be analyzed in clinical trials for their effectiveness. Currently, there are virus-directed inhibitors intended to block the IC activity of the E protein SARS-CoV-2 with encouraging results from in vitro assays [32,43,44].

Studies conducted on SARS-CoV-1 showed that recombinant viruses lacking the E protein were attenuated in diverse animal models, leading to a significant reduction in pulmonary edema in mice and lower mortality rates than wild-type viruses [40,45]. Attempts to use these viruses as live-attenuated vaccines have been evaluated both with complete E-deleted recombinant viruses (rSARS-CoV-ΔE) or partial deletions of the protein. Immunization with rSARS-CoV-ΔE induced high serum neutralizing antibodies levels in hamsters and almost completely protected mice against lethal challenge with mouse-adapted SARS-CoV. Notably, these recombinant viruses induce both humoral and cellular responses [46,47,48].

E protein contains a short linear motif (SLiM) in its carboxy-terminal sequence, which has been identified as a PDZ-binding motif (PDZbm) [21]. This motif is defined by the four last aa in a protein and is classified into three types according to the consensus sequence: X-S/T-X-Φ for type I, X-Φ-X-Φ for type II, and X-D/E-X-Φ for type III (where X is any amino acid, and Φ is a hydrophobic amino acid) [49]. The E protein of SARS-CoVs contains a class II PDZbm corresponding to the DLLV sequence (Figure 1), which allows the E protein to interact with diverse host PDZ (PSD-95/DLG/ZO-1) domain-containing proteins, favoring viral dissemination as explained below [21,50]. In the case of the most pathogenic CoV, MERS, it bears an E protein with a type II PDZbm (DEWV) capable of interacting with a greater number of PDZ proteins compared to SARS-CoVs. (Table 2).

PDZ proteins constitute a large family of scaffold proteins that are highly conserved among species and are characterized by the presence of at least one PDZ domain. These proteins govern multiprotein complex formation and localization to diverse subcellular sites; thus, they are involved in a variety of fundamental cellular functions, such as cell trafficking, proliferation, signal transduction, and polarization. PDZ domains are the most common protein–protein interaction domains in the human proteome. They are composed of approximately 90 amino acids, and their 3D folding forms a hydrophobic cavity that allows interactions with PDZbm in their ligands (Figure 1D) [56,57]. As mentioned above, PDZ domains participate in multiple PPIs related to diverse fundamental cellular functions; outstandingly, several viral pathogens have been described to express PDZbm motifs that usurp host PDZ-dependent interactions for their own purposes [50,58]. Highly pathogenic CoVs are among these viruses, and in the case of the SARS-CoVs, the E protein expresses a PDZbm which is recognized as a major determinant of virulence; that is, the PDZbm-dependent interactions established by E with host PDZ proteins play a major role in SARS-CoV-2 virulence [59].

Recombinant SARS-CoVs with mutated PDZbm in the E protein or lacking the full-length protein generally restore the PDZbm sequence or generate a novel chimeric gene to restore the absence of PDZbm after several cell passages [21,60]. This reinforces the idea that by mimicking endogenous ligands, PDZbm-dependent interactions established by E with host PDZ proteins play a determining role in SARS-CoVs virulence and pathogenicity. The envelope proteins of SARS-CoV-1 and SARS-CoV-2 are 96% similar, maintaining conserved the IC-forming region, as well as the PDZbm. PALS1 (protein associated with Lin seven-1) was the first PDZ protein to be described as a target for PDZbm of the E protein of SARS-CoV-1 [51,52,59]. PALS1 is involved in the maintenance of apicobasal epithelial cell polarity, and its targeting by the E protein destabilizes the integrity of the epithelial layer, promoting viral dissemination [52]. Novel experimental assays have demonstrated that this interaction is strengthened by the E protein of SARS-CoV-2, and it has been proposed that this increased affinity contributes to the increased virulence of SARS-CoV-2 versus SARS-CoV-1 [52,53]. Syntenin is another PDZ interactor of the E protein of SARS-CoV-1 that induces a high production of inflammatory cytokines by activating p38 MAPK; this may increase the permeability of the epithelial barrier in favor of viral dissemination [21]. Moreover, the PDZbm of the E protein of SARS-CoV-2 interacts with the tight junction protein ZO-1, another PDZ protein involved in the integrity of the respiratory tract epithelium layer [54,55]. Although this interaction needs to be confirmed in in vivo assays, it seems that such an interaction would compromise epithelial integrity, similar to the interaction of E with PALS1. The E protein is conserved in more than 98% of SARS-CoV-2 isolates, including different emerging VOCs [33]. To date, several PDZ proteins have been identified as E protein targets (Table 2), most of which have been identified by physicochemical methods, such as affinity chromatography, equilibrium binding titration by Förster Resonance Energy Transfer (FRET), calorimetric assays, and yeast two-hybrid [55,61]. It is important to address whether these interactions occur in vivo and the cellular functions they alter. Functional and structural analyses of specific PDZ-dependent interactions are relevant for understanding these virus–human PPIs and their potential for intervention. SLiMs are commonly expressed in intrinsically disordered regions, and the C-terminal PDZbm of the E protein of highly pathogenic CoVs (SARS-CoV-1, MERS, and SARS-CoV-2) is expressed within a more flexible structure than that of the less virulent CoVs causing common cold (Figure 1B); the high flexibility of these PDZbms in highly pathogenic CoVs has been proposed to contribute to their interaction with a large number of host PDZ proteins and to cause more severe diseases [62]. Recent experimental data appear to support this hypothesis. In recent work, a series of recombinant SARS-CoV-1 in which E protein PDZbm was substituted with those from high or low pathogenic human CoVs were generated [63]. Infection of mice with these recombinant viruses demonstrated that PDZbm of low pathogenic CoVs partially attenuated SARS-CoV-1-induced pathology reducing lung cellular infiltration and edema in infected mice. In contrast, mice infected with recombinant viruses carrying PDZbms of the most virulent human CoVs developed extensive cellular infiltration to the lungs, edema and pronounced weight loss leading to the death of infected mice [63]. Furthermore, it has been demonstrated that the most pathogenic MERS displays the largest array of PDZ interactors (Table 2).

In addition, although PDZbm is conserved between SARS-CoV-1 and 2, it appears that the SARS-CoV-2 E protein binds more host PDZ proteins than SARS-CoV-1, probably with higher affinity, as has been suggested for PALS1. This may imply that amino acid substitutions/deletions near PDZbm might affect the structure, and IC activity and/or confer more flexibility to the SARS-CoV-2 E protein, thus defining its PDZ interactome (Table 2) [61,62]. Notably, the E proteins of the three highly pathogenic CoVs share at least five PDZ interactors, leading to the possibility of designing a pan-coronavirus inhibitor.

## 5. PDZ-Dependent PPI Inhibitors

PDZ-dependent PPIs have been widely studied as therapeutic targets, and both endogenous and pathogen-derived PDZbms have shed light on important functional interactions that are useful for drug design. Most inhibitors are directed to the interaction interface between the PDZ hydrophobic cavity and the PDZbm ligand, acting as competitive agonists to prevent the PDZ-PDZbm interaction [64]. Notably, many PDZ-dependent interactions have been identified as targets of small molecules or peptides that bind with high affinity, some of which have been assayed for their therapeutic function, mainly against cancer and nervous system disorders, such as neuropathic pain or ischemic stroke. At least one endogenous PDZ-PDZbm PPI inhibitor, a small molecule, is commercially available for cancer treatment, and other small molecules and peptides are being assayed in clinical trials [65]. Blocking PDZ-dependent interactions is a promising strategy for developing specific treatments not only for COVID-19 but against diverse viral infections. Furthermore, recently revealed PDZ-dependent interactions that are conserved among highly pathogenic CoVs may serve to design pan-viral inhibitors for further emerging coronavirus epidemics and pandemics.

## 6. Conclusions

Mapping virus–host PPIs is important for understanding viral pathogenicity and revealing the fundamental host proteins hijacked for successful viral replication. The E protein of CoVs is one of the most abundantly expressed in infected cells and is an important virulence factor of highly pathogenic CoVs. Furthermore, within the E protein sequence, the PDZ-binding motif has been recognized as a virulence determinant by itself; hence, identification of its target proteins in infected cells is mandatory to gain insight into the viral pathogenic mechanisms and, concomitantly, to document potential therapeutic targets. Targeting host PDZ proteins seems to contribute significantly to the highly pathogenic CoVs life cycle; thus, targeting these virus–host interactions may help control airway epithelial damage and viral dissemination during COVID-19. Interfering the host–virus interface could be a comprehensive strategy to reduce resistance emergence, as the virus is less likely to mutate at such specific interacting moieties. It is tempting to consider the blockage of PDZ-dependent interactions of the highly pathogenic CoV E protein with host PDZ proteins as a promising method for the current COVID-19 pandemic control. Additionally, since PDZbm is a conserved motif among distinct CoV strains, it would be appealing to consider the generation of pan-CoV inhibitors using viral PDZbm-based PPIs.

## Figures and Tables

**Table 2:** PDZ interactors with the E protein of the most pathogenic coronaviruses.

Coronavirus	SARS-CoV-1	SARS-CoV-2	MERS	Reference
**PDZbm**	**Type 2: DLLV**	**Type 2: DLLV**	**Type 3: DEWV**	
Human PDZ interactors	HTRA1 LNX2 MLLT4 PALS1 PARD3 PDZD2 PTPN13 SDCBP TJP1	HTRA1 LNX2 MAST2 MLLT4 NHERF1 PALS1 PARD3 PTPN13 RADIL SDCBP SNX27 TJP1	APBA1 ARHGAP21 FRMPD4 GIPC2 GORASP1 GORASP2 HTRA1 HTRA3 INADL LAP2 LNX2 LRRC7 MAGI1 MAGI2 PARD3 PDZD2 PTPN13 RGS3 RIMS1 RIMS2 SDCBP SIPA1L2 SNX27 SYNPO2L TJP1 TJP3	[21,51,52,53,54,55]

## Data Availability

No new data were created or analyzed in this study. Data sharing is not applicable to this article.
